# Zebrafish as a model to investigate the CRH axis and interactions with DISC1

**DOI:** 10.1016/j.coemr.2022.100383

**Published:** 2022-10

**Authors:** Helen Eachus, Soojin Ryu, Marysia Placzek, Jonathan Wood

**Affiliations:** 1 Living Systems Institute and College of Medicine and Health, University of Exeter, Exeter, United Kingdom; 2 Institute of Human Genetics, University Medical Center, Johannes Gutenberg University Mainz, Mainz, Germany; 3 School of Biosciences and Bateson Centre, University of Sheffield, Sheffield, United Kingdom; 4 Sheffield Institute for Translational Neuroscience and Department of Neuroscience, University of Sheffield, Sheffield, United Kingdom

**Keywords:** Zebrafish, CRH, CRF, HPA axis, HPI axis, Hypothalamus, DISC1

## Abstract

Release of corticotropin-releasing hormone (CRH) from CRH neurons activates the hypothalamo–pituitary–adrenal (HPA) axis, one of the main physiological stress response systems. Complex feedback loops operate in the HPA axis and understanding the neurobiological mechanisms regulating CRH neurons is of great importance in the context of stress disorders. In this article, we review how *in vivo* studies in zebrafish have advanced knowledge of the neurobiology of CRH neurons. Disrupted-in-schizophrenia 1 (DISC1) mutant zebrafish have blunted stress responses and can be used to model human stress disorders. We propose that DISC1 influences the development and functioning of CRH neurons as a mechanism linking DISC1 to psychiatric disorders.

## Highlights

• CRH neurons in the hypothalamus regulate the stress response of mammals and zebrafish.
• The development and function of CRH neurons are tightly regulated.
• DISC1 may influence CRH neuron development and function.
• Understanding CRH neuron regulation is important in the context of stress disorders.

## Introduction: zebrafish as a model to investigate the CRH axis

**Figure 1:**
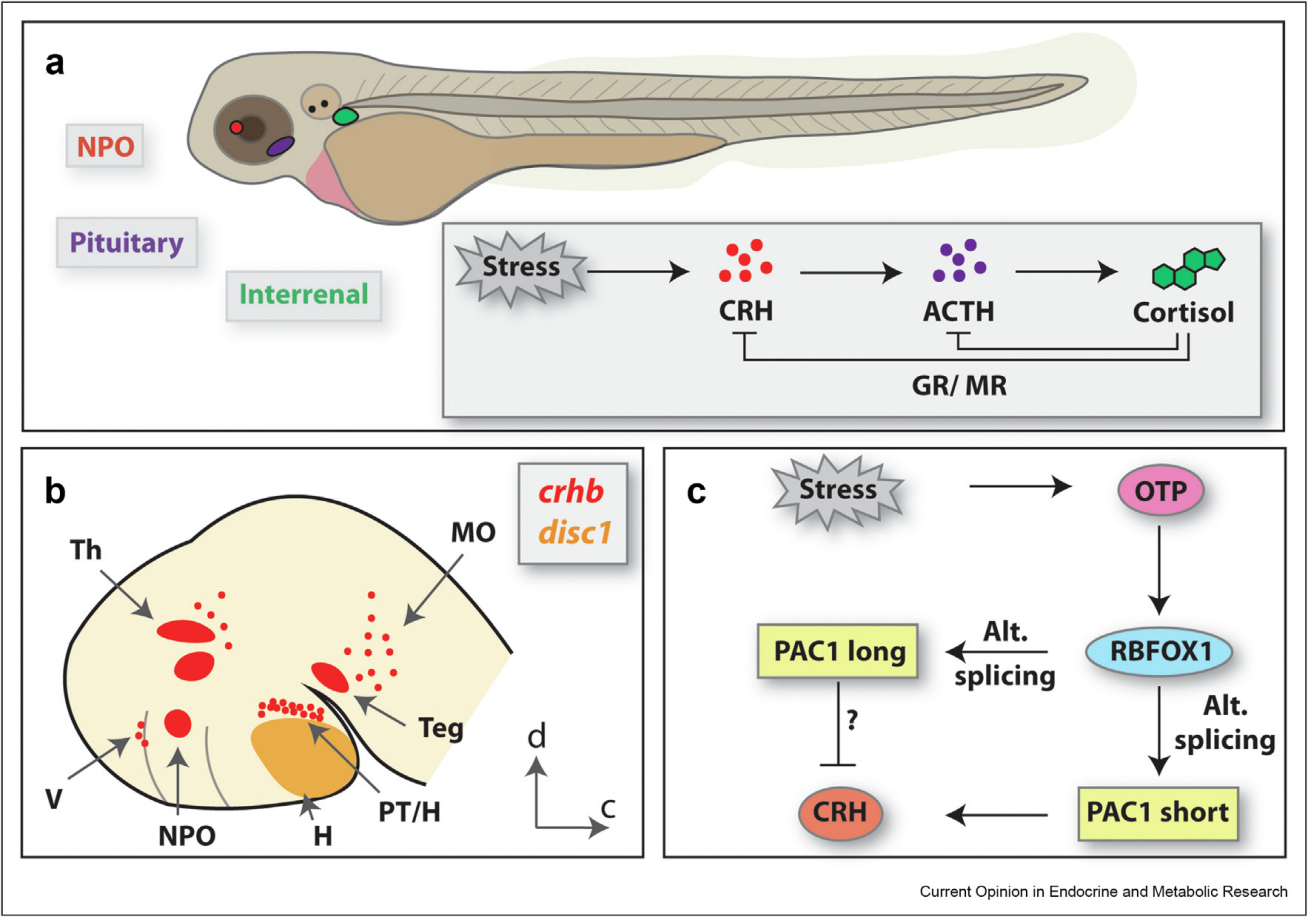
**The hypothalamo–pituitary–interrenal (HPI) axis in zebrafish, its regulation and cell types.** (**a**) A schematic showing the HPI axis in a zebrafish larva with its key anatomical centres and molecular pathways. Environmental stress leads to release of Crh from the neurosecretory preoptic area (NPO) of the hypothalamus. Crh induces the synthesis and release of ACTH from the pituitary gland, which initiates synthesis of the key stress hormone cortisol in the interrenal gland. Negative feedback to *crh* and ACTH (*pomc*) occurs via cortisol binding to its receptors GR (glucocorticoid receptor) and MR (mineralocorticoid receptor). (**b**) A cartoon of a 2–3 dpf zebrafish brain showing the locations of *crhb* and *disc1* expression. *crh-*expressing cells are found in the telencephalon (V), posterior tuberculum and tuberal hypothalamus (PT/H), neurosecretory preoptic area (NPO), thalamus (Th), tegmentum (Teg), rostral hindbrain (MO) [45]. Within the 2–3 dpf brain, *disc1* expression is observed in the hypothalamus (H), especially the tuberal region [39]. d, dorsal, c, caudal. (**c**) A schematic depicting the regulation of the CRH-mediated stress response. Adapted from Amir-Zilberstein et al., 2012 [18]. Stress activates Otp-mediated transcription of *crh*, and of *rbfox1* which regulates alternative splicing of the *pac1* gene. Otp and the PAC1-short variant both upregulate *crh* transcription, whilst the PAC1-long isoform is required for termination of *crh* transcription.

Corticotropin-releasing hormone (CRH), also known as corticotropin-releasing factor, is a 41-amino acid peptide. While produced in diverse tissues in many parts of the body, the most studied role of CRH is its function as a releasing hormone involved in the hypothalamo–pituitary–adrenal (HPA) axis (or hypothalamo–pituitary–interrenal, (HPI) axis in fish)-mediated stress response. When the HPA axis is activated by a stressor, CRH is released from parvocellular neurons of the hypothalamic paraventricular nucleus (PVN) which project to the median eminence; here, CRH enters the portal system and is transported via small capillary vessels to the anterior pituitary gland. In fish, HPI axis activation induces the secretion of CRH from neurons of the neurosecretory preoptic area (NPO), the fish equivalent of the mammalian PVN [1]: these cells send direct neuronal projections to the rostral pars distalis of the anterior pituitary gland (Figure 1a). In the corticotroph cells of the anterior pituitary gland, CRH binds to its receptor. In both fish and mammals, two receptors exist, CRH receptor 1 and CRH receptor 2 (CRHR1 and CRHR2, respectively), with CRHR1 being the primary receptor in the fish HPI axis [2]. Receptor binding then initiates the synthesis and release of ACTH (adrenocorticotropic hormone), which reaches the adrenal gland or interrenal gland (the fish counterpart of the adrenal gland) via the circulation. ACTH binds its receptor MC2R (melanocortin 2 receptor) in the steroidogenic cells of the interrenal gland, which initiates synthesis of cortisol, the key stress hormone in both fish and humans (Figure 1a).

In mouse, lesion studies and genetic studies show that CRH neurons are pivotal to the stress response [3, 4, 5]. However, the difficulty in accessing the mammalian hypothalamus, which lies deep within the brain, has limited such studies. The zebrafish brain is much smaller and lends itself well to *in vivo* neurophysiological experiments. Zebrafish provide a genetically tractable high-throughput system for investigating neurodevelopmental and neurophysiological mechanisms underpinning behavioural phenotypes [6, 7, 8]. In this review, we will discuss the zebrafish CRH system, focusing on the hypothalamic CRH neurons. Further, we discuss interactions of CRH with disrupted-in-schizophrenia 1 (DISC1), a risk factor for psychiatric disease, for which animal models indicate alteration of the CRH system.

## CRH neurons in the zebrafish hypothalamus

CRH neurons in the zebrafish are distributed throughout the brain, in a reasonably well conserved manner between zebrafish and mammals [9]. In mammals, CRH is encoded by a single gene. In teleosts, two CRH genes, *crha* and *crhb*, evolved via genome duplication [10]. The *crha* and *crhb* genes encode 162 and 132 amino acid polypeptides, respectively, sharing 44% identity and 56% similarity. *crhb* is considered orthologous to the mammalian *CRH* gene, and has been far more widely studied than *crha*, whose expression is restricted to the periventricular hypothalamus [10]. *crhb* is first expressed at around 24 h post fertilization (hpf) and from 3 days post fertilization (dpf) can be detected in multiple regions of the brain, including the subpallium, preoptic region, posterior tuberculum, hypothalamus, ventral thalamus and hindbrain, as well as in the retina [11,12] (Figure 1b).

In zebrafish*, crh*-expressing neurons in the preoptic region are found in the NPO. Within this region, *crh*-expressing neurons form a dense intermingled cluster with other neurons that express *oxytocin* (*oxt*), *arginine vasopressin* (*avp*), *proenkephalin a* (*penka*), *neurotensin* (*nts*) and *somatostatin* (*sst1.1*) [1,13]; whilst neurons that express *cholecystokinin* (*cck*), *proenkephalin b* (*penkb*) and *vasoactive intestinal peptide* (*vip*) are found in separate NPO subregions [13]. Within the NPO cluster, *crh* shows a high degree of co-expression with *avp*, which similarly to *crh* also stimulates ACTH secretion, and shows a low degree of co-expression with *penka* and *penkb* [13]. Outside of the NPO, *crh*-expressing cells can be found in other regions of the hypothalamus [11,12], especially in the tuberal region, as is also the case in mouse [9]. Here, their functions are less clearly understood.

## Functional studies in fish support a role for CRH neurons of the NPO in stress regulation

Work in zebrafish has revealed how CRH neurons of the NPO respond to acute stress exposure *in vivo* in an intact animal [14]. Using two-photon calcium imaging, vom Berg-Maurer et al. showed that stressor exposure in the form of salinity or pH alteration leads to an increased pool of active CRH NPO cells, via recruitment of previously inactive cells. The number of responsive CRH NPO cells also increased with stressor intensity and those cells also exhibited an increase in the number of Ca^2+^ events. These data suggest that CRH cell activity is tightly regulated and varies in accordance with stressor intensity, to ensure that the HPA axis response is proportionate to the threat severity.

Building on this, a subsequent study has investigated the role of various NPO neuropeptides in the behavioural response to stress, using molecular, imaging and computational techniques [15]. To a greater extent than some other NPO peptidergic populations, *crh*-expressing neurons showed specialisation in their responsiveness to specific threats. For example, many *crh*-expressing neurons responded purely to heat, salinity or acidity, rather than responding to a combination of threats. Interestingly, while ablation of either *crh* or *oxt*-expressing neurons had no effect on the behavioural response to a threat, combined ablation of both significantly reduced the behavioural response to the aversive stimuli, suggesting that these separate neuronal clusters work together to promote stress-induced behaviour. Subsequent experiments indicated that the *oxt* and *crh*-expressing NPO clusters are largely glutamatergic and, in addition to projecting to the pituitary, both project to a specific set of spinal-projecting neurons in the brainstem, which are essential for the motor response to stress. The observed behavioural responses to the aversive stimuli were observed over a very short time period, suggesting a role for CRH NPO neurons in rapid locomotor responses to various stressors.

Recently, the CRH system has been studied *in vivo* in zebrafish using a CRISPR *crhr1* knockout (KO) fish [16]. *crhr1* KO larvae were unable to respond to an acute stressor, that is, endogenous cortisol levels did not rapidly increase following stress exposure as would be observed in wild types. Interestingly, *crhr1* KO larvae also exhibited altered behaviour in a light–dark locomotion assay in which larvae typically freeze during the light period and swim freely during the dark period [16]. During the dark phase, *crhr1* KO larvae exhibited some hypoactivity compared with wild types at the 15 min and 60 min time points. Further, during the light phase, stress exposure induced hyperactivity in wild type larvae, but this was not observed in *crhr1* KO larvae. However, further experiments suggested that stress-induced hyperactivity is likely mediated predominantly by downstream effects of cortisol, rather than by CRH itself. Further work with this model may shed light on whether CRH regulates other stress-induced behaviours in fish, independently of cortisol, as is observed in rodents [17].

## Regulation of CRH expression in the stress response

Regulation of CRH is critical to mount a proper stress response. Zebrafish experiments have implicated the homeodomain-containing protein orthopedia (Otp) in transcriptional regulation of *crh* during the stress response. Zebrafish have two othologues of OTP, *otpa* and *otpb*. *otpa* null zebrafish do not upregulate *crh* expression following acute stressor exposure [18]. Chromatin immunoprecipitation (ChIP) experiments subsequently revealed that Otp protein is recruited to the zebrafish *crh* promoter region following exposure to a stressor. In a similar manner, Otp is also recruited to the promoter of the *a2bp1* (more commonly known as the splicing regulator *rbfox1*) gene following stress. RBFOX1 is known to regulate the alternative splicing of neuronal *Pac1*, which encodes the receptor for the pituitary adenylate cyclase activating peptide. The authors demonstrated that the short *pac1* variant is required for normal upregulation of *crh* transcription following stress, while the *pac1-hop* (long) mRNA isoform is required for normal termination of *crh* transcription during the recovery phase following stress (Figure 1c), as well as normal regulation of initial and recovery phase levels of endogenous cortisol. Furthermore, the *pac1-hop* (long) variant was found to be important for the behavioural response to an acute stressor in zebrafish larvae. Together, the experiments support that alternative splicing of *pac1* is required for stressor-induced regulation of *crh* transcription, the HPA axis and behaviour, at least partly via Otpa-mediated transcriptional control of *rbfox1*.

In addition to the identification of molecules that appear to acutely regulate expression of *crh* in the context of HPI axis regulation, animal models are also useful in identifying molecules that may regulate the development of CRH neurons, which in turn may affect their function in the HPA axis. Classic knockout studies in rodents have identified multiple players involved in developmental regulation of CRH neurons. Terminal differentiation of PVN neurons, including those that express *Crh*, requires *Otp* and *Single-minded 1* (*Sim1*) in mouse [19], in conjunction with *Aryl-hydrocarbon receptor nuclear translocator 2* (*Arnt2*) [20]. *Brn2* is also required downstream for *Crh* expression [19]. Studies in zebrafish suggest that transcriptional regulation of *crh* in development is conserved, since loss of both *otp* paralogs in zebrafish leads to a complete loss of the NPO *crh* cluster [21]. Additionally, the development of *crh-*expressing neurons in the embryonic zebrafish NPO is also dependent on transcription factors *arnt2* and *sim1a* [22].

Zebrafish studies have also identified a number of novel potential regulators of *crh* in development. Zebrafish mutants for chemokine-like gene *sam2* (*samdori 2*) exhibit anxiety-like behaviour in the novel tank, scototaxis and shoaling assay tests, and have a significant increase in *crh* expression in the preoptic region [23]. *Sam2*-KO mice also exhibited anxiety and fear behaviour and in the PVN SAM2 appears to regulate the frequency of GABAergic inputs onto CRH neurons. Conversely, adult knockout fish for Down's syndrome-associated gene *dyrk1aa* have reduced expression of preoptic *crh* following exposure to social isolation [24]. The authors argue that this may indicate a low responsiveness to stress in the context of social isolation, with an implication for human stress-associated disorders.

## Zebrafish models of human stress disorders: CRH-DISC1 connection

One of the best-described zebrafish models of a human stress disorder is the DISC1 (Disrupted-in-schizophrenia 1) zebrafish. *DISC1* encodes a multifunctional scaffold protein that shows a plethora of protein–protein interactions. It was first identified at the breakpoint of a balanced (1; 11) (q42.1; q14.3) translocation segregating with mental illness in a large Scottish family more than twenty years ago [25]. In the decade following its discovery, evidence accrued for *DISC1* being a risk factor in a range of psychiatric illnesses, and for it having roles in neurodevelopment and neural signalling pathways [26]. Although the relevance of *DISC1* to mental illness in the wider population remains contentious [27], *DISC1* mutant and transgenic mice have face validity as models of human psychiatric disease, bringing an understanding of the pathophysiological mechanisms underlying mental illness [28]. *Disc1* mutant mice show wide-ranging behavioural and neural phenotypes [28, 29, 30]. These include a defective HPA axis, which manifests as alterations in the stress response. Attenuated reactivity of the HPA axis was reported in a transgenic mouse strain carrying an inducible mutant human *DISC1* gene prenatally challenged with polyI:C, a preclinical model of schizophrenia [31]. In a second study, a converse phenotype was reported: plasma corticosterone levels were shown at elevated levels after mild isolation stress in adolescent transgenic mice carrying a mutant human *DISC1* gene [32]. These apparently conflicting results may reflect, amongst others, the different stress paradigms utilised, differences in the expression profile of mutant human *DISC1* through use of different promoters, or the complex feedback loops that operate in the HPA axis.

Given the ease of genetic manipulation, the small transparent brain, the opportunity to perform high-throughput behavioural analysis and that the HPA axis is conserved amongst vertebrates, the zebrafish offers a powerful system in which to study the role of DISC1 in the HPA axis. Zebrafish have a single *DISC1* orthologue on chromosome 13 in a region showing synteny with human chromosome 1 [33]. The RNA-binding arginine-rich motif (ARM) [34] and coiled-coil domains are evolutionarily conserved [35] suggesting that these regions are crucial for DISC1 function. While DISC1 has a multitude of protein interaction partners, a study which characterised the endogenous DISC1 interactome in iPSC-derived neural progenitor cells and astrocytes revealed that poly(A) RNA-binding and centrosomal dynamics are core (i.e. common to both cell types) DISC1 functions [36]. These functions can at least in part be attributed to the evolutionarily conserved regions of *DISC1* [34,37,38]. DISC1 is therefore likely to have conserved functions in neurodevelopment and neural signalling.

**Figure 2:**
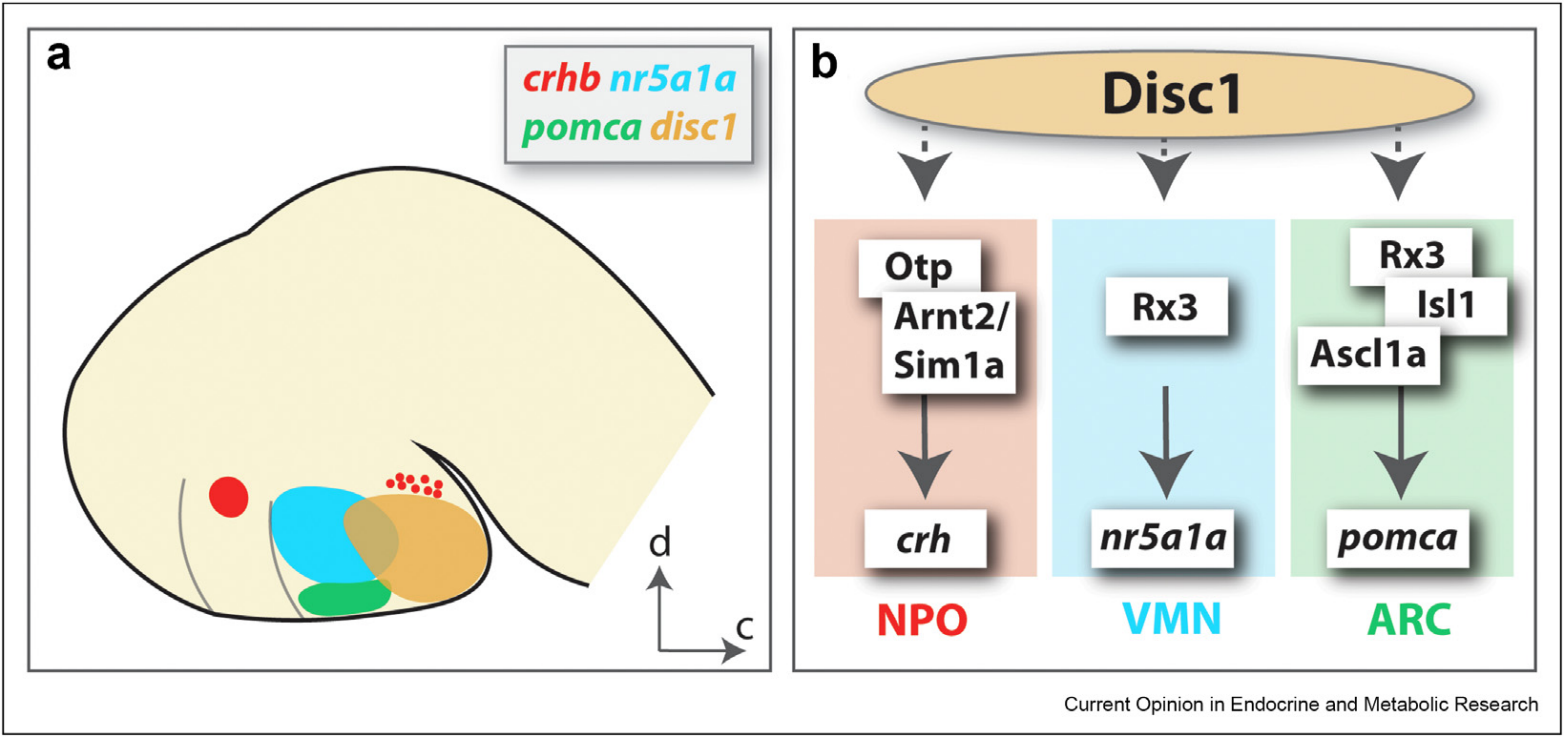
**Regulation of zebrafish hypothalamic development by Disc1.** (**a**) A cartoon depicting the locations of potential Disc1-regulated neurons in the 2–3 dpf zebrafish hypothalamus. *crh-*expressing neurons are located in the NPO and tuberal region of the hypothalamus (as well as other brain regions, not shown). *nr5a1a* neurons are located in the ventromedial hypothalamic nucleus (VMN) [46]. *pomca* neurons are located in the arcuate nucleus (as well as the pituitary gland, not shown) [46]. d, dorsal, c, caudal. (**b**) Schematic showing possible regulation of specific hypothalamic nuclei by Disc1 in zebrafish. The development of *crh-*expressing neurons in the NPO is known to be regulated by Otp, Arnt2 and Sim1a [22]. Rx3 is known to regulate the development of *nr5a1a-*expressing neurons in the VMN [46]. Rx3, Ascl1a and Isl1 are known to regulate the development of *pomca-*expressing neurons in zebrafish [40,46, 47, 48]. Since expression of NPO *crh*, VMN *nr5a1a* and ARC *pomca* neurons is altered in *disc1* mutant zebrafish [39], Disc1 may act as an upstream regulator of these developmental pathways.

In embryonic zebrafish, *disc1* expression is prominent in the ventral diencephalon, including the tuberal hypothalamus [33] (Figure 1b). Here, *disc1*-expressing cells lie close to *crh*-expressing neurons, and to neurons that express *nr5a1a* and *pomca* (the latter, in regions that are functionally equivalent to the mammalian ventromedial nucleus and arcuate nucleus) (Figure 2a). Homozygous *disc1* mutants have altered expression of *crh* in the tuberal hypothalamus, but also show altered expression of *crh* in the preoptic hypothalamus [39]. The effects of *disc1* mutation are dynamic across the early life period: increased *crh* expression is observed in the preoptic NPO region at embryonic stages, but decreased *crh* expression is detected in the larval hypothalamus in *disc1* mutants. The reduction in *crh-*expressing neurons at larval stages correlates with a blunted behavioural and endocrine response to acute stress exposure in mutant larvae, consistent with the attenuated HPA axis reactivity described previously [8]. Given the essential role of *crh* in stress regulation and the altered stress response in *disc1* mutant zebrafish [39], it seems likely that *disc1* regulates the HPA axis via *crh*. At present the mechanism by which *disc1* regulates development of hypothalamic *crh* neurons is unknown.

In addition, *disc1* mutants show altered differentiation of *pomca* and *nr5a1a*-expressing neurons. Potentially, these effects reflect an alteration in the maintenance of *rx3*-expressing (retinal homeobox 3) hypothalamic progenitors in the *disc1* mutant fish. Both *pomca* and *nr5a1a* expressing neurons arise from *rx3*-expressing progenitors [46], which co-express *disc1* in the tuberal hypothalamus [39] (Figure 2b). As yet, the mechanism responsible for the altered numbers of *crh*-expressing neurons in *disc1* mutants is unclear. Whilst *rx3*-expressing progenitor cells are not known to give rise to *crh*-expressing neurons, neither a lineage analysis of *rx3*-expressing progenitors, nor a comprehensive analysis of *crh*-expressing neurons following *rx3* knockout has been performed in zebrafish to date (see Ref. [40] for an initial analysis). Alternatively, *disc1* may regulate *crh* neuron development by acting on one of the known *crh* regulators such as *otp* [18,41], *arnt2* and *sim1a* [22]*, brn2* or LIM homeobox 2 *(lhx2)* [42] (Figure 2b), or via an as yet unknown pathway. In addition to regulation of *crh* neuron development, *disc1* may also mediate the HPA axis via alteration of *crh* neuron function, but this remains to be determined.

## Future perspectives

Recent studies in zebrafish have indicated that activity of CRH neurons in response to stress is complex and tightly regulated, with diverse aspects of CRH neuron activity varying with respect to the threatening stimulus [14,15]. Measuring neuronal activities in zebrafish using techniques such as *in vivo* calcium imaging is a powerful tool to tease out the complex function of CRH neurons in an intact animal. For example, calcium imaging in *disc1* mutant fish with and without stressor exposure could provide insights into whether *disc1* regulates activity of CRH neurons *in vivo*. Similarly, calcium imaging of CRH neurons in animals reared under different contexts such as early life stress exposure could reveal how development shapes the stress response. Single cell RNA sequencing (scRNAseq) studies in mammals indicate that there are multiple subsets of hypothalamic *Crh*-expressing cells, even within the preoptic region [43,44]. As such, studies in zebrafish, a simpler model organism with a smaller brain, yet a high degree of molecular conservation can be used to interrogate the development and function of these small populations of cells. Indeed, scRNAseq of *crh*-expressing cells in *disc1* mutant fish could be used to tease out specific sub-population differences in CRH neurons resulting from a lack of *disc1* function, as well as identifying molecules linking *disc1* with CRH neuron development and function.

## Conflict of interest statement

The authors declare the following financial interests/personal relationships that may be considered as potential competing interests. SR holds a patent, European patent number 2928288 and US patent number 10, 080, 355: ‘A novel inducible model of stress.’ The remaining authors declare that they have no known competing financial interests or personal relationships that could have appeared to influence the work reported in this paper.
